# Serum and Urinary Neutrophil Gelatinase-Associated Lipocalin Levels as Early Markers of the Renal Function in Patients With Urinary Stone-Induced Hydronephrosis

**DOI:** 10.3389/fsurg.2022.843098

**Published:** 2022-03-14

**Authors:** Tiancheng Xie, Hongmin Zhou, Yuchen Gao, Xiao Xu, Xudong Yao, Xulin He, Yunfei Xu

**Affiliations:** Department of Urology, Shanghai Tenth People's Hospital, Tongji University, Shanghai, China

**Keywords:** NGAL, renal function, hydronephrosis, urinary stones, marker

## Abstract

**Introduction:**

Urinary stones cause hydronephrosis, which leads to kidney function impairment. The serum creatinine level is frequently used as a marker of kidney function. However, in some patients with hydronephrosis, it does not reflect the kidney function changes in the early stages of kidney stone disease. Neutrophil gelatinase-associated lipocalin (NGAL) is a novel indicator of the kidney function. Previous NGAL-related research has focused on its role in acute kidney injury. This study aimed to determine the usefulness of NGAL as an early marker of the kidney function in patients with urinary stones and hydronephrosis.

**Methods:**

Eighty-five patients with urinary stones who were admitted to the Shanghai Tenth People's Hospital (USP group) and 65 healthy volunteers (NC group) were recruited. Blood and urine samples collected from the study participants were evaluated using an enzyme-linked immunosorbent assay to determine the NGAL concentration. Data on the height, weight, age, medical history, and blood and urine findings were collected. Computed tomography data were collected from the USP group.

**Results:**

Compared to in the NC group, NGAL levels were significantly elevated in the USP group (*P* < 0.001). However, no significant differences in the NGAL levels were observed among the USP group members with different degrees of hydronephrosis. Furthermore, no significant between-group differences in the creatinine level or the estimated glomerular filtration rate were observed. The areas under the receiver operating characteristic curves for the serum and urinary NGAL levels with hydronephrosis were 92.03 and 99.54%, respectively. The areas under the receiver operating characteristic curves for the serum and urinary NGAL levels with kidney stones were 85.05 and 91.89%, respectively.

**Conclusion:**

NGAL is a sensitive indicator of hydronephrosis secondary to urinary stones.

## Introduction

Urinary stone disease is very common (1). Urinary stones form in the kidneys and bladder, and can be classified as either upper or lower stones based on their location. Natural discharge of some urinary stones may occur, depending on their size, shape, and location. However, some urinary stones persist in their particular urinary tract location. When stones block the renal pelvis junction or the ureter, acute complete obstruction or chronic incomplete obstruction may occur. Hydronephrosis is caused by the stenosis of the urethral lumen and the obstruction of the urethra in any part of the kidney to the external orifice of the urethra, and gradually damages the renal parenchyma and affects the kidney function. At present, there are some different standards for the classification of hydronephrosis in the world (2–4).

Currently, serum creatinine level is one of the main markers of kidney function (5–8). Creatinine is a small molecule that can be filtered through the glomerulus, and is rarely absorbed by the renal tubules. Almost all creatinine is excreted through the urine. When renal insufficiency occurs, creatinine accumulates in the body, causing toxicity. The estimated glomerular filtration rate (eGFR) reflects the kidney function (9). It is a measure of the volume of blood that the glomerulus can filter per minute, and is used to evaluate the ability of the kidney to remove metabolic waste from the body (10, 11). However, there are certain limitations of using serum creatinine as an indicator. In particular, the serum creatinine level does not reflect the status of kidney function in a timely and accurate manner (12, 13). Increases in the serum creatinine levels may manifest clinically only when most of the kidney has suffered from pathological damage and the glomerular filtration rate has decreased by more than 50%. Therefore, the serum creatinine level does not accurately indicate changes in kidney function at an early stage of disease.

In Kjeldsen et al. (14) discovered a novel 25-kD protein while studying the 92-kD MMP-9 protein in neutrophils. This protein was named neutrophil gelatinase-associated lipocalin (NGAL). Subsequent studies have shown that NGAL is expressed in trace amounts by the neutrophils and certain epithelial cells (such as those in the renal tubules). In ischemic or nephrotoxic kidney injury, NGAL is expressed in greater quantities by the kidneys and released into the urine and plasma. NGAL levels increase within 2 h of a renal tubular epithelial cell injury, making it an early and sensitive biomarker of renal injury. At present, research on NGAL is mainly focused on its role in acute kidney injury (AKI), including severe infections, obstructive pulmonary disease, and so on (15–18).

Many diseases affect kidney function; however, most do not do so as quickly as AKI. Nonetheless, early detection of kidney function remains important (18). Currently, most hospitals use creatinine as a marker of kidney function. In our clinic, we observed that in case of patients with urinary stones, changes in the serum and urine creatinine levels are not obvious in those with a small amount of hydronephrosis. Whether NGAL can reflect the changes in kidney function caused by urinary stones and hydronephrosis remains unknown. Therefore, we carried out a prospective clinical study to determine whether NGAL may be used as an early indicator of changes in the kidney function secondary to urinary stone-induced hydronephrosis.

## Methods

### Research Subjects

In this study, 85 patients who were hospitalized at the Tenth People's Hospital of Tongji University (between July 2020 and December 2020) for urinary stones were enrolled and categorized into the USP group. In addition, 65 volunteers who underwent an ultrasound examination of the urinary system to rule out the possibility of hydronephrosis and urinary stones were enrolled as healthy controls in the NC group. We took the short diameter of the renal pelvis cavity under ultrasound as greater than 1 cm as the standard for hydronephrosis. The following inclusion criteria were applied: no history of diseases that may impair renal function, such as hypertension and diabetes; no history of acute or chronic renal impairment; no history of kidney surgery; and first identification of kidney stones and hydronephrosis. Patients with urolithiasis underwent Computed Tomography (CT) and the CT data were collected. The following data were collected from the study participants: age, weight, height, eGFR, and medical history. The body mass index (BMI) for each participant was also calculated from their height and weight. This cohort study was following STOBE guidelines and approved by the ethics committee of Shanghai Tenth People's Hospital (approved number was 21K105), and informed consent was obtained from all patients who participated in the study.

### Laboratory Assays

From each participant, 2 mL of blood and 5 mL of urine were collected for further analysis. The blood samples were allowed to stand at room temperature for 2 h; they were then centrifuged, and the serum was extracted. The serum and urine samples were stored at −80°C for future analysis. Serum creatinine and urea nitrogen levels were measured at the Central Laboratory of the hospital. The serum and urinary NGAL concentrations were measured using the Human NGAL/Lipocalin-2 ELISA Kit (RJ14849, Shanghai Renjie Biotechnology Co. Ltd.).

### Statistical Analysis

Statistical analyses were performed using the SPSS 16.0 statistical package, and charts were made using GraphPad Prism 7. If the measurement data were normally distributed, means and standard deviations and the *t*-test were used to perform the intergroup comparisons. If the measurement data did not follow a normal distribution, the medians (interquartile ranges) and the rank-sum test were used to perform the intergroup comparisons. Count data were compared using the chi-square test. Statistical significance was set at *p* < 0.05.

## Results

### Clinical Characteristics of the Study Subjects

Compared with the NC group (serum NGAL level 59.06 ± 12.22 ng/ml, urinary NGAL level 67.08 ± 11.99 ng/ml), the USP group had significantly elevated serum NGAL level (89.20 ± 15.64 ng/ml, *P* < 0.001) and urinary NGAL level (110.92 ± 14.74 ng/ml, *P* < 0.001). However, no significant between-group differences were observed in the sex (NC 52 male:13 female, USP 67 male:18 female, *P*-value 0.860), height (NC 167.49 ± 7.32 cm, USP168.35 ± 7.37 cm, *P*-value 0.481), weight (NC 68.96 ± 11.15 kg, USP 69.52 ± 11.77 kg, *P*-value 0.763), BMI (NC 24.54 ± 3.35, USP 24.43 ± 3.21, *P*-value 0.846), serum creatinine levels (NC 77.80 ± 27.03 umol/L, USP 83.36 ± 29.81 umol/L, *P*-value 0.234), serum urea nitrogen concentrations (NC 5.38 ± 2.30 mmol/L, USP 6.85 ± 7.76 umol/L, *P*-value 0.102), CKD-EPI (NC 92.30 ± 23.82 mL/(min^*^1.73 m^2^), USP 89.36 ± 23.23 mL/(min^*^1.73 m^2^), *P*-value 0.451). The characteristics of the study participants are summarized in Table 1.

**Table 1:** Clinical characteristics of the subjects.

	**USP (*n* = 85)**	**Control (*n* = 65)**	***P*-value**
Male/Female	67:18	52:13	0.860
Height (cm)	168.35 ± 7.37	167.49 ± 7.32	0.481
Weight (kg)	69.52 ± 11.77	68.96 ± 11.15	0.763
BMI	24.43 ± 3.21	24.54 ± 3.35	0.846
Serum NGAL (ng/ml)	89.20 ± 15.64	59.06 ± 12.22	<0.001
Urinary NGAL (ng/ml)	110.92 ± 14.74	67.08 ± 11.99	<0.001
Serum creatinine (umol/L)	83.36 ± 29.81	77.80 ± 27.03	0.234
Serum urea nitrogen (mmol/L)	6.85 ± 7.76	5.38 ± 2.30	0.102
CKD-EPI [ml/(min*1.73 m^2^)]	89.36 ± 23.23	92.30 ± 23.82	0.451

### Correlations Between the Serum and Urinary NGAL Levels and the Clinical Characteristics

Correlations between the serum NGAL levels and the clinical characteristics (including the age, height, weight, BMI, serum creatinine level, and serum urea nitrogen concentration) are shown in Table 2. Correlations between the urinary NGAL levels and the clinical characteristics (including the age, height, weight, BMI, serum creatinine, and serum urea nitrogen concentration) are shown in Table 3.

**Table 2:** Correlations between serum NGAL levels and clinical characteristics.

	**USP (*****n*** **=** **85)**		**NC (*****n*** **=** **65)**		**All (*****n*** **=** **150)**	
	**r**	***P***	**r**	***P***	**r**	***P***
Age	−0.123	0.264	0.110	0.381	0.004	0.960
Height	0.018	0.867	−0.216	0.085	−0.005	0.954
Weight	0.121	0.268	−0.126	0.317	0.040	0.628
BMI	0.145	0.185	−0.008	0.951	0.047	0.564
Serum creatinine	0.072	0.512	0.225	0.072	0.156	0.057
Serum urea nitrogen	0.074	0.499	−0.102	0.418	0.118	0.150
eGFR	0.039	0.720	−0.301	0.015	−0.106	0.196

**Table 3:** Correlations between Urinary NGAL levels and clinical characteristics.

	**USP (*****n*** **=** **85)**		**NC (*****n*** **=** **65)**		**All (*****n*** **=** **150)**	
	**r**	***P***	**r**	***P***	**r**	***P***
Age	0.045	0.681	−0.201	0.109	0.014	0.862
Height	−0.147	0.179	0.051	0.685	0.012	0.888
Weight	−0.170	0.119	−0.016	0.897	−0.039	0.637
BMI	−0.120	0.274	−0.057	0.652	−0.064	0.439
Serum creatinine	−0.113	0.304	0.212	0.090	0.084	0.307
Serum urea nitrogen	−0.093	0.396	0.010	0.937	0.064	0.434
eGFR	0.085	0.439	−0.127	0.312	−0.051	0.532

### Comparison of the Serum and Urinary NGAL Levels

**Figure 1:**
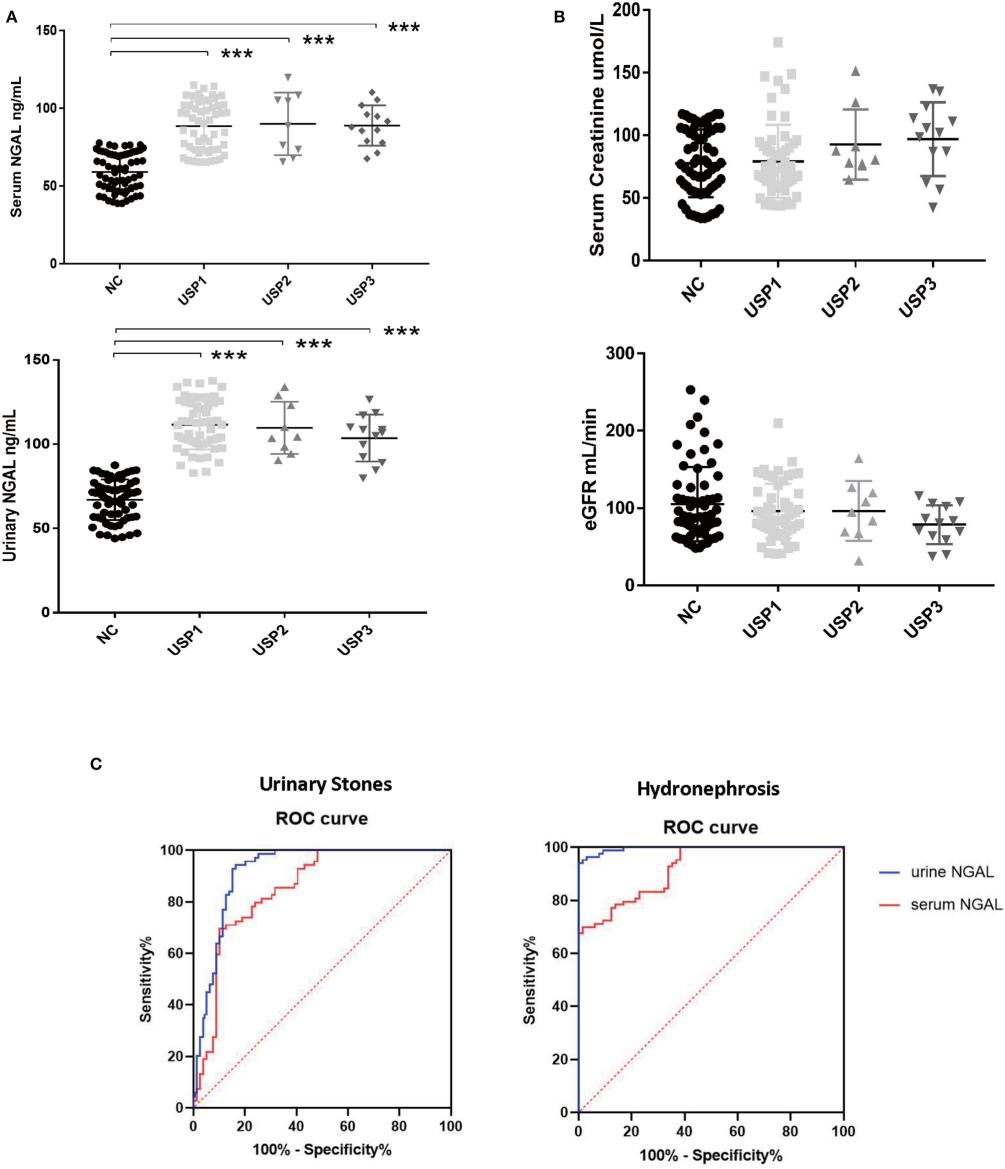
Data analysis results. **(A)** Differences in the serum and urinary NGAL levels among the different groups. **(B)** Comparison of the creatinine concentrations and estimated glomerular filtration rates among the different groups. **(C)** Serum and urinary NGAL levels in the diagnosis of urinary stones and hydronephrosis. ***P < 0.001.

In view of the need to study the relationship between different degrees of hydronephrosis and NGAL in adult patients with urinary calculi, we think that there is no suitable classification of hydronephrosis. We sorted the hydronephrosis data of 85 patients from small to large, and divided them into three groups with one third as the node, mild hydrops group (USP1), moderate hydrops group (USP2), and severe hydrops group (USP3). Results indicated significant differences in the serum and urinary NGAL levels between the healthy control group and each USP group (*P* < 0.001). However, there were no significant differences in the serum/urinary NGAL levels among the USP subgroups (Figure 1A).

### Comparison of the Creatinine Concentrations and the EGFR Between the Different Groups

Although some trends in were observed, no significant between-group differences in creatinine concentrations and CCR were observed (Figure 1B).

### Serum and Urinary NGAL in the Diagnosis of Hydronephrosis Caused by Kidney Stones

A receiver operating characteristic (ROC) curve of the serum and urinary NGAL levels with hydronephrosis and kidney stones was created. Results indicated that for hydronephrosis, the areas under the ROC curve for the blood and urine NGAL levels were 92.03% (95% confidence interval [CI]: 88.03–96.03%, *p* < 0.0001) and 99.54% (95% CI: 98.94–100%, *p* < 0.0001), respectively. For kidney stones, the areas under the ROC curve for the blood and urine NGAL levels were 85.05% (95% CI: 78.79–91.3%, *p* < 0.0001) and 91.89% (95% CI: 87.22–96.57%, *P* < 0.0001), respectively (Figure 1C).

## Discussion

In this study, experimental findings showed that NGAL is an extremely sensitive indicator of changes in the kidney function secondary to urinary stone-induced hydronephrosis when the creatinine levels did not change significantly. This is consistent with findings indicating that the hysteresis phenomena are associated with creatinine. Early-stage hydronephrosis due to urinary stones induces stress in the renal tubular epithelial cell and enhances the secretion of NGAL. However, NGAL levels do not reflect the different degrees of the effects of hydronephrosis on the kidney function. This finding may be related to the sensitivity by which NGAL indicates the presence of the stones. Consistent with prior findings, ROC curve findings showed that NGAL is highly correlated with hydronephrosis and kidney stones, and it can be used to predict the presence or absence of kidney stones and hydronephrosis. Our findings showed that mild hydronephrosis caused by kidney stones was sufficient to significantly increase the NGAL expression.

NGAL, also known as the human neutrophil lipoprotein, is a monomer composed of 178 amino acid residues. It functions as a 25-kDa monomer, self-polymerizes to form a 46-kDa homodimer, and polymerizes with MMP-9 to form a 135-kDa heterodimer (14, 19). NGAL is a recently identified lipocalin, which is synthesized in the bone marrow during myogenesis. It is exported from the bone marrow and stored in the neutrophil granules (20). It is expressed in non-hematopoietic organs and tissues, such as the colon, trachea, lung, and kidney epithelium (21). Stressed renal epithelial cells secrete NGAL (22, 23).

NGAL is highly expressed in animal kidneys and is released into the urine after ischemia or nephrotoxic injury (24). *NGAL* is one of the most rapidly upregulated genes after ischemic AKI in animals (24). It was noted in a study that after ischemia and nephrotoxic AKI, the urinary concentration of renal-induced NGAL increased several fold over a short period. Studies have shown that the plasma and urinary NGAL levels in critically ill patients are associated with AKI severity (25). Therefore, NGAL is considered a promising biomarker for AKI; yet, its clinical application as a biomarker may not be limited to AKI. Recent studies have shown that NGAL levels are associated with inflammation, immune response, chemotaxis, signal transduction, and occurrence and development of many types of tumors (15, 25).

Renal stones are caused by the abnormal accumulation of crystalline substances (such as calcium, oxalic acid, uric acid, and cystine) in the kidneys. It is a common disease that occurs more frequently in men than in women, and is particularly predominant in young men. Stones can occur in any part of the urinary system, but their formation often initiates in the kidneys. Kidney stones tend to be located in the renal pelvis or calyces, and can be discharged to the ureter or bladder (26, 27). Nearly all ureteral stones originate in the kidneys. Kidney stones often cause hydronephrosis, in which the kidney is swollen; this impairs the kidney function. Patients with hydronephrosis are often asymptomatic for extended periods, until renal colic, an abdominal mass, or waist swelling occurs. Hydronephrosis often influences kidney function at the early stages of the disease; however, its symptoms are not obvious and are often ignored. Currently, the serum creatinine level is the most frequently used marker of renal function. However, due to the hysteresis of serum creatinine, it does not accurately reflect the early temporal changes in the renal functioning. NGAL is a more sensitive indicator of the renal function; however, prior to this study, whether NGAL reflects the changes in the early renal function secondary to urinary stone-induced hydronephrosis remained unknown.

At the same time, we also note that there are some limitations in this study. Since this is the first time we have conducted a cohort study on the clinical significance of NGAL in the diagnosis of hydronephrosis caused by urinary calculi, the sample size of this study is small, the detection of urine creatinine level is lacking, and the analysis of NGAL and prognosis in patients with urinary calculi is lacking. We will further improve it in the follow-up cross-sectional study.

## Conclusion

NGAL is a very sensitive indicator of hydronephrosis caused by urinary stone.

## Data Availability Statement

The raw data supporting the conclusions of this article will be made available by the authors, without undue reservation.

## Ethics Statement

The studies involving human participants were reviewed and approved by the Ethics Committee of Shanghai Tenth People's Hospital. The patients/participants provided their written informed consent to participate in this study.

## Author Contributions

TX, XH, and YX: conception and design. HZ, YG, XX, and XH: data acquisition. HZ and YG: analysis and interpretation of data. TX, HZ, YG, and XX: statistical analysis. XX: drafting of the manuscript. XY: revision of the manuscript. YX: supervision. All authors contributed to the article and approved the submitted version.

## Funding

This study was funded by National Natural Science Foundation of China (Nos. 81971371 and 82101671) and 2019 Scientific Research Funds of Shanghai Tenth People's Hospital (No. 04.03.19.043).

## Conflict of Interest

The authors declare that the research was conducted in the absence of any commercial or financial relationships that could be construed as a potential conflict of interest.

## Publisher's Note

All claims expressed in this article are solely those of the authors and do not necessarily represent those of their affiliated organizations, or those of the publisher, the editors and the reviewers. Any product that may be evaluated in this article, or claim that may be made by its manufacturer, is not guaranteed or endorsed by the publisher.
